# A Study of a GNSS/IMU System for Object Localization and Spatial Position Estimation

**DOI:** 10.3390/s25226968

**Published:** 2025-11-14

**Authors:** Rosen Miletiev, Peter Z. Petkov, Rumen Yordanov

**Affiliations:** 1 Faculty of Telecommunication, Technical University of Sofia, 1000 Sofia, Bulgaria; pjpetkov@tu-sofia.bg; 2 Faculty of Electronics, Technical University of Sofia, 1000 Sofia, Bulgaria; yordanov@tu-sofia.bg

**Keywords:** IMU, GNSS, Kalman filter, quaternions, Allan Variance

## Abstract

Today, navigation systems are commonly used in a variety of applications such as autonomous vehicles, image stabilization, object detection and tracking, and virtual reality (VR) or artificial reality (AR) systems. These systems require not only the precise location but also the accurate tracking of the orientation of rigid bodies moving in a three-dimensional (3D) space. This study introduces the integration of GNSS and a 10DoF IMU system to solve the navigation task and calculation of the object position, attitude, and heading. As the location and the attitude calculations require different states but use the same data from the INS sensors, the sensor data fusion in two Kalman filters is proposed. As the filters’ performance is critical, according to the initial states, we study in detail the Allan Variance and normal distribution parameters of three different MEMS IMU sensors. The GNSS system performance and statistics are examined using two commercial and three proposed single or dual-band GNSS antennas. An experimental study is conducted, and the KF output of the heading angle is compared with other sources.

## 1. Introduction

The Global Navigation Satellite System (GNSS) has been the most common positioning and navigation system for decades [1], but its performance depends fully on signal propagation, and it provides a continuous navigation solution only if enough satellites are visible. To ensure robust and continuous tracking, another navigation system, e.g., inertial navigation system, is proposed to be employed to calculate the object location and motion when the GNSS service is not available [2].

The contemporary world needs more precise navigation, and an accurate spatial position calculation is required, especially in many civilian applications, including smart driving, unmanned aerial vehicle (UAV) control, and transportation tracking. The Global Navigation Satellite System (GNSS) provides the geographic coordinates, speed vector, and accurate time of a moving object, while an autonomous inertial navigation system (INS) using the inertial measurement unit (IMU), such as a linear accelerometer and angular rate sensor, determine the object orientation, such as the attitude and heading values. This attitude and heading reference system (AHRS) is very important in applications such as autonomous vehicles, image stabilization, object detection and tracking, and AR/VR systems. In most cases, these two independent navigation systems may be integrated to calculate the position, as the GNSS may be corrupted or lost, while the INS navigation system accuracy diverges with time due to the inertial error accumulation.

The GNSS errors are not always statistically independent; however, assuming independence simplifies the mathematical models used to estimate the position and perform error analysis. The last generation GNSS receivers may update the position up to 20 Hz, due to the very complex computation task of the correlation processes, and the first position fix may be delayed for up to 45 s. In addition, the GNSS signals may be completely lost in many situations—entering tunnels, underground garages, etc. In such cases, an autonomous INS may solve the navigation task, but it requires another navigation system such as a GNSS [3], wheel speed sensor (WSS) [4], Doppler velocity log (DVL) baseline system [5], or terrain map [6] to correct its accuracy. The Kalman filter (KF) technique has been widely implemented for GNSS/INS integrated systems to overcome this navigation problem for a limited period of time [7,8,9,10]. A detailed analysis of the existing Kalman filters is presented in [11].

A Kalman filter is also used for spatial orientation on the basis of the noisy sensor data from the inertial measurement units (IMUs) with other sources such as a GNSS or magnetometers to provide a more accurate and reliable estimate of an object’s orientation in 3D space, often expressed using Euler angles or quaternions [12]. By modeling sensor biases and using a predictive process, the filter combines these diverse inputs, weighted by their estimated reliability, to produce a stable and precise orientation, which is crucial for navigation, robotics [13], UAVs, and motion tracking systems [14]. The sensor fusion techniques based on the Kalman filter models can even be implemented in the 32-bit ARM^®^ Cortex™-M0+ microcontrollers, which are integrated in the BNO055 [15] or BNO08X [16] sensors optimized for use with virtual reality (VR) or artificial reality (AR) controller systems. The calculated values of the quaternion and the Euler angles are directly readable from the application microcontroller. In our previous work [17], we conducted a comparison analysis of the heading accuracy of the sensor fusion software integrated in BNO055 and the developed Kalman filter.

The organization of this article is as follows. In Section 1, the system design is presented for the object location and spatial position estimation. The block diagram and the schematics are also described. Section 2 lists the MEMS sensors selection for the GNSS/IMU system design, according to their noise parameters and output data rate (ODR) values. In this section, the theory of calculation of the Euler angles is shown based on the Kalman filter. The tuning of this Kalman filter is an essential step of this work; therefore, the noise parameters are measured and set in the measurement covariance matrix R and process noise covariance matrix Q. Section 3 presents the study of GNSS sub-systems using different antennas, such as commercial and proposed ones. The results are provided, and the performance of the IMU and GNSS sub-systems is calculated and compared in Section 4 and Section 5. Finally, the conclusions are presented.

## 2. System Design

### 2.1. Hardware Description of the GNSS/IMU System

A state-of-the-art inertial measurement unit (IMU) consists of a tri-axial accelerometer, a tri-axial gyro, and a tri-axial magnetic sensor, henceforth referred to as an integrated nine degrees-of-freedom (DoF) IMU. The additional barometric sensor may be added to form a complete 10 DoF navigation system. In addition, some temperature sensors may be installed; however, contemporary MEMS sensors have an integrated sensor for temperature compensation.

**Figure 1:**
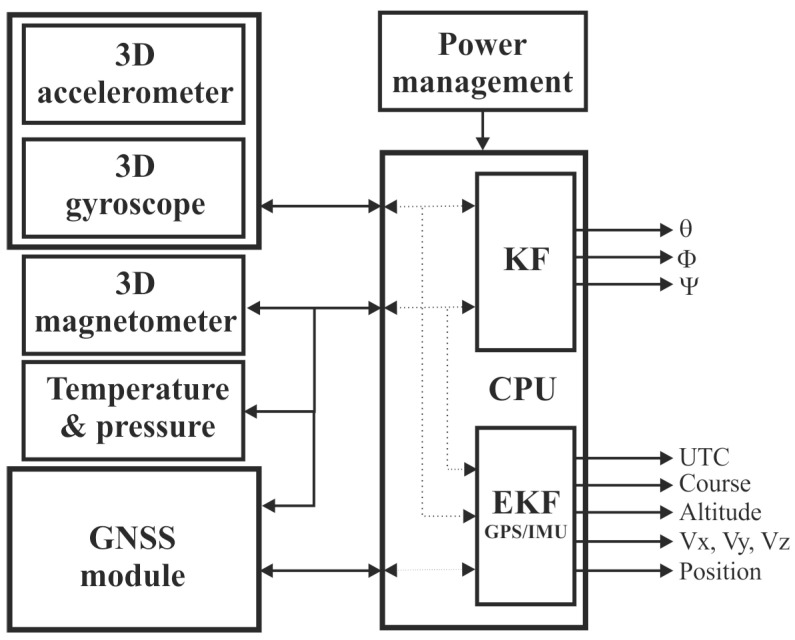
System design for object location and spatial position estimation.

The system design (Figure 1) comprises a 10 DoF navigation system and GNSS receiver for estimation of the Euler angles (pitch, roll, and yaw) and the object position and speed vector (course, altitude, and speed magnitude). The object position and orientation are calculated using two independent Kalman filters, as this article only discusses the design and tuning of the Kalman filter regarding calculating the spatial position.

The IMU systems may calculate the spatial object’s position and its movement direction with a refresh rate over 400 Hz; however, the position calculation is a recursive process, which can lead to an expanded error accumulation. The integration of both navigation systems may significantly reduce their drawbacks. Therefore, the navigation system was distinguished with 10 degrees of freedom (DoF) and contains a three-axis linear accelerometer sensor, three-axis angular rate sensor, a three-axis magnetometer, and a barometric sensor as an altitude sensor, where the accelerometer’s output data rate (ODR) should be high enough to minimize the integration errors.

**Figure 2:**
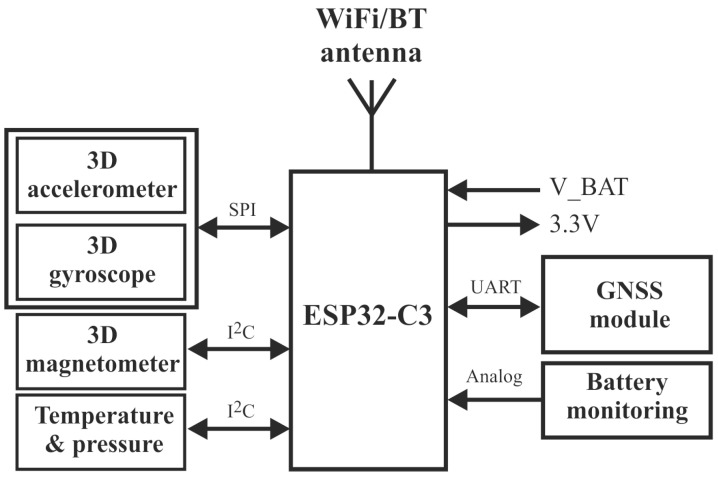
GNSS/IMU system integration block diagram.

The system block diagram is shown in Figure 2. The proposed system is based on EPS32-C3 SoC, which includes a 32-bit microcontroller up to 160 MHz and WiFi6/BT5 connectivity, a six DoF inertial measurement unit (IMU), an 18-bit three-axis magnetometer, and a 24-bit resolution barometer as an altitude sensor, to design a complete 10 DoF navigation system with a u-blox GNSS module.

As the board is powered by providing 5 V at Vin pin, the CMOS low dropout (LDO) voltage regulator is used, as RT9080 by Richtek, to convert the input voltage to 3.3 V@600 mA for the GNSS receiver and the ESP32-C3 power supply. The selection of this LDO regulator should provide a minimum 500 mA output current to ESP32-C3 SoC, and its very low drop-out voltage is only 310 mV@600 mA load, while the LDO output is stable with ceramic and tantalum output capacitors.

Most IMUs are connected to 32-bit microcontrollers [16,17] and external communication devices. The selection of the ESP32-C SoC is based on the integration of a 32-bit core with WiFi6/BT5 connectivity, with secure IoT applications. For example, the ESP32-C3 SoC includes a 32-bit core RISC-V microcontroller with a maximum clock speed of 160 MHz and low power modes, while the ESP32-C6 SoC includes a low-power (LP) 32-bit RISC-V processor, which can be clocked up to 20 MHz, and supports 2.4 GHz Wi-Fi 6, Bluetooth 5 (LE), and the 802.15.4 protocol (Thread/Zigbee).

The ESP32-C SoC selection is also based on the integrated FPU (floating point unit) that may perform 32-bit single-precision operations, providing powerful computing capabilities needed for the Kalman filter implementation, and its high memory resources, such as 400 KB SRAM, 384 KB ROM, and a built-in 4 MB FLASH. The support of USB communication for programming and debugging is also an advantage of this system on a chip (SoC). If the CPU resources are not enough, due to the complexity of the EKF, which depends on the filter states, this SoC may be easily upgraded to a dual-core ESP32-S3.

**Figure 3:**
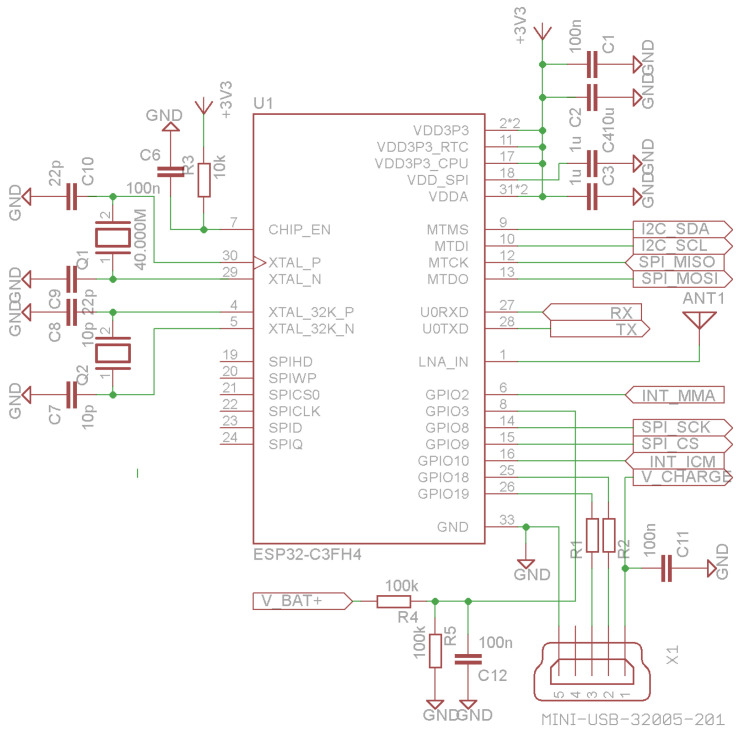
ESP32-C3 SoC connection schematic.

The ESP32-C3’s Real-Time Clock (RTC) is also used and is connected to the external 32.768 kHz crystal for improved timing accuracy, especially in deep sleep modes, while the main clock uses the 40.000 MHz crystal oscillator (Figure 3).

### 2.2. MEMS Sensor Selection for the GNSS/IMU System Study

The low-cost inertial MEMS sensors (for example, the automotive grade sensors) are distinguished with a higher zero-rate offset, drift over temperature, and output noise, which make their measurements less accurate and reduce their performance, compared with other inertial sensors. The analysis of the commercial MEMS inertial sensors (for linear and angular acceleration) for navigation purposes covers sensors from several well-known manufacturers such as ST Microelectronics, TDK, and Bosch as follows:

- BMI270 (Bosch);
- ICM-42688-P (TDK);
- LSM6DSR (ST Microelectronics).

The reasons for choosing the specified sensors are as follows:

- Built-in three-axis sensors for measuring linear and angular acceleration in one housing: six DoF with the required resolution of 16-bit;
- Compatibility of input/output ports;
- High data refresh rate (ODR ≥ 1 kHz);
- Low noise level;
- Small dimensions (2.5 × 3.0 × 1.0 mm);
- Built-in temperature sensors for internal data compensation;
- SPI/I2C interfaces for communication.

The above shown sensors are selected and compared with the following criteria, which are the most important for the navigation purposes:

- Digital resolution;
- Zero-rate offset;
- Output noise density;
- Output data rate.

A comparison study of the parameters of the proposed sensors is shown in Table 1 and Table 2 for the linear accelerometers and angular rate sensors, respectively.

The sensor parameter comparison tables show that the ICM-42688-P sensor has better zero-rate offsets and higher output data rates compared with the other sensors, which is very important for navigation purposes, as the numerical integration errors are proportional to the output offset and Δt3, where Δt denotes the reciprocal of the output data rate.

We also analyzed 3D MEMS magnetometers with applicability in a high-accuracy 10 DoF navigation system, and the MEMS sensor MMC5983MA from MEMSIC was selected, distinguished by its maximum resolution of 18 bits and minimum noise of 0.4 mG total RMS, which allows determination of the course with an accuracy of ±0.5° [21].

The analysis of high-resolution (24-bit) atmospheric pressure sensors with applicability in a 10 DoF navigation system is related to the selection of a sensor with the maximum resolution of 24 bits to determine the altitude of the object based on the atmospheric pressure value. For this purpose, the LPS22HB piezoresistive sensor from ST Microelectronics was selected, which, in addition to the necessary sensitivity and noise level, also features a built-in temperature sensor for internal data compensation, which enables achieving an accuracy of ±0.1 hPa for the pressure measurement [22]. The barometer data are used in the EKF for the GNSS/IMU integration and should be discussed further.

Based on the sensor selection, three different 10 DoF navigation systems were investigated. The configuration of the sensors on the PCB board was as shown in Table 3.

**Figure 4:**
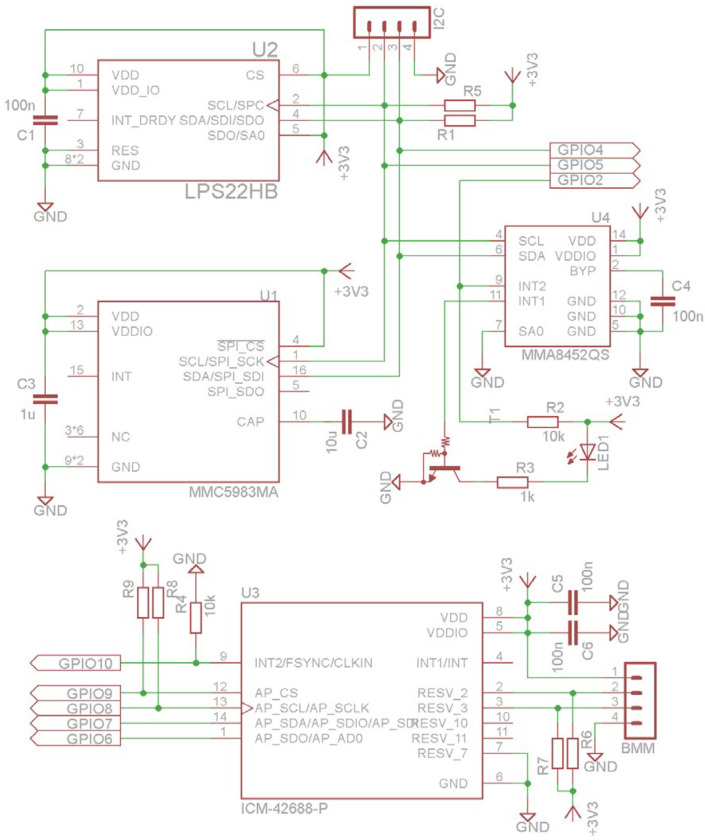
IMU system design.

To achieve the best sensor performance (high sampling rate, simultaneous sensor reading, etc.), the MEMS sensors were connected to the different communication interfaces, as shown in Figure 2 and Figure 4. The linear accelerometer and angular rate sensor were connected to the ESP32-C3 SoC via SPI interface @ 20 Mbps, while the magnetometer and the barometer were connected to the host via an I2C interface @ 400 kbps. The second 3D linear accelerometer MMA8452Q was also connected to the I2C interface, as it is configured in motion or transient detection mode. The sensor register data are read when an interrupt signal is detected at the sensor alarm output. The interrupt pins INT1 and INT2 (Figure 4) were connected in parallel in the software configuration and trigger an interrupt at the GPIO2 pin of the ESP32 chip. The other connections to the ESP32 GPIO pins are also shown in Figure 4.

As the different MEMS sensors, shown in Table 3, may support different output data rates (ODR), the system should be configured to use one common ODR. All sensors (accelerometer, gyroscope, magnetometer, and barometer) have to use the same ODR, as the data are read by the ESP32-C3 SoC using an interrupt routine driven by the 6 DoF MEMS sensor INT output (Figure 4). On the basis of the ICM-42688-P sensor ODR characteristics, shown in Table 4, the update rate of 50 Hz was selected.

### 2.3. Calculation of the Euler Angles Based on the Kalman Filter

**Figure 5:**
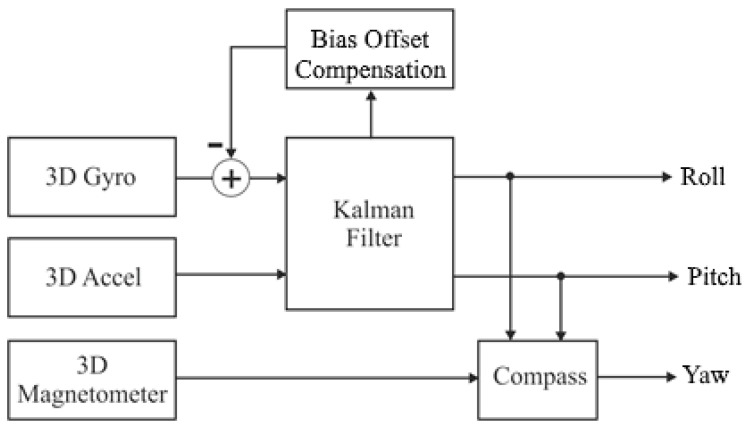
Block diagram of the linear Kalman filter model.

The data from the sensors, such as accelerometers, gyroscopes, and magnetometers, are used to calculate the objects’ spatial position based on a linear Kalman filter model, which is used to combine the data from the individual sensors. The corresponding diagram is shown in Figure 5.

The linear model of the Kalman filter is represented by Equations (1) and (2) [23]:

(1) $$x_{k+1}={Ax}_{k}{+B\cdot u_{k}+w}_{k},$$

(2) $$y_{k}=H\cdot x_{k}+z_{k},$$

where *x* is a system state; *A*, *B*, and *H* are the state, input, and measurement matrices, respectively; *y* is a measured output; *u* is a known input; and *w* and *z* are process and measurement noise, respectively, with the following mean and covariance:

(3) $$E\left\{w_{k}\right\}=E\left\{z_{k}\right\}=0,$$

(4) $$E\left\{w_{k}\cdot z_{k}^{T}\right\}=0,$$

(5) $$E\left\{w_{k}\cdot w_{k}^{T}\right\}=\left\{\begin{array}{c} Q_{k},k=i\\ 0,k\neq i \end{array},\right.$$

$$E\left\{z_{k}\cdot z_{k}^{T}\right\}=\left\{\begin{array}{c} R_{k},k=i\\ 0,k\neq i \end{array},\right.$$

where $E\left\{\cdot \right\}$ denotes the expectation function; *Q* and *R* are the covariance matrix of the process noise and measurement errors, respectively.

The Kalman filter operation algorithm is based on two main actions: a priori and a posteriori estimations. In the first step, called the time update, the values of vector *x* are predicted, and the covariance matrix $P_{k}^{-}$ is estimated according to the following equations:

(6) $$x_{k}^{-}=Ax_{k-1}+Bu_{k-1},$$

(7) $$P_{k}^{-}=AP_{k-1}A^{T}+Q,$$

where $x_{k}^{-}$ is the predicted state estimation, $P_{k}^{-}$ is the predicted covariance matrix, $P_{k-1}$ is the update estimated covariance matrix, and $x_{k-1}$ is the updated state estimation.

The Kalman filter update states may be described as follows [24]:

(8) $$K_{k}=P_{k}^{-}\cdot H^{T}\cdot {\left(HP_{k}^{-}\cdot H^{T}+R_{k}\right)}^{-1},$$

(9) $$x_{k}=x_{k}^{-}+K_{k}\cdot (y_{k}-Hx_{k}^{-}),$$

(10) $$P_{k}=\left(I-K_{k}\cdot H\right)\cdot P_{k}^{-},$$

where $K_{k}$ is the Kalman filter gain, $x_{k}$ is the updated state estimation vector, $y_{k}$ is the measurement vector, $P_{k,}$ is the updated estimated covariance matrix, and I is the identity matrix.

The object’s spatial position may be represented by the Euler angles *Φ*: pitch, *θ*: roll, and *ψ*: yaw (Figure 5), or by quaternions [25] to avoid the “gimbal lock” problems and singularity. The pitch and roll value calculations use the input and measurements vectors, which are defined in Equations (11) and (12), respectively:

(11) u=[ω],

(12) y=[α],

where α denotes an angle, and ω is the angular rate.

Taking x as the state vector, matrices *A*, *B*, and *H* are given as follows:

(13) $$x=\left[\begin{array}{c} \alpha \\ q_{b} \end{array}\right],$$

(14) $$A=\left[\begin{array}{cc} 1 & -T\\ 0 & 1 \end{array}\right],$$

(15) $$B=\left[\begin{array}{c} T\\ 0 \end{array}\right],$$

(16) $$H=\left[\begin{array}{cc} 1 & 0 \end{array}\right],$$

where *T* denotes the sampling interval, and $q_{b}$ is a gyroscope bias, which is included in the state vector giving the algorithm the possibility of correcting the angular velocity value.

In the case of orientation evaluation,

(17) $$\alpha_{k}=\alpha_{k-1}+\left(\omega_{k}-q_{b,k-1}\right).T,$$

(18) $$q_{b,k}=q_{b,k-1}.$$

As the pitch and roll angles are estimated, the calculation of the magnetic field components may be accomplished as follows:

(19) $$M^{l}=R_{m}.M^{b}\;,$$

where *M* denotes the magnetic field components’ (*M_x_*, *M_y_*, *M_z_*) matrix in a local horizontal frame (*l*) and body coordinate frame (*b*), and $R_{m}$ denotes the rotation matrix:

(20) $$R_{m}=\left[\begin{array}{ccc} cos\theta & sin\Phi sin\theta & -cos\theta sin\Phi \\ 0 & cos\Phi & sin\Phi \\ sin\theta & -sin\theta cos\Phi & cos\Phi cos\theta \end{array}\right].$$

Therefore, the orientation precision of the magnetic orientation systems not only depends on the calibration validity of the magnetometers but also is closely related to the errors of the tilt angles. The magnetic heading *ψ* may be calculated as follows:

(21) ψ=−arctanMylMxl, (Mxl>0;Myl≤0)π2, (Mxl=0;Myl<0)π−arctanMylMxl,(Mxl<0)3π2,(Mxl=0;Myl>0)2π−arctanMylMxl,(Mxl>0;Myl>0).

The calculated magnetic heading angle may be converted to the geographic north using the magnetic declination based on a topographic map or the use of an online tool such as NOAA’s Declination Calculator.

### 2.4. Tuning of the Kalman Filter

A major advantage of the Kalman filter is that it performs the most optimal state estimation and provides an estimate of the state covariance matrix. The matrix *Q*, representing the system noise covariance, and *R*, which is the measurement noise covariance matrix are as follows:

(22) $$Q=\left[\begin{array}{cc} T^{2}\sigma_{\alpha }^{2} & 0\\ 0 & \sigma_{\alpha }^{2} \end{array}\right],$$

(23) $$R=\left[\sigma_{\omega }^{2}\right],$$

where $\sigma_{\alpha }$ represents the orientation measurement noise, and $\sigma_{\omega }$ represents the angular rate measurement noise.

The accuracy and behavior of a Kalman filter relies on the values placed in the different covariance matrices. The measurement noise is modeled and determined by the measurement noise covariance matrix R. High values in the matrix R may not correct the IMU sufficiently, while small values cause the system to rely more on the sensor data than the model [26].

There are different methods to model and estimate the inertial sensor stochastic errors, such as the Autoregressive (AR) model [27,28,29], the Gauss–Markov (GM) model [30], and the Allan Variance (AV) [31,32]. These methods are verified on modeling inertial sensors errors [33].

A common technique used to quantify various noise sources in inertial sensor data is called the Allan Variance [34]. The Allan Variance (AV) identifies the stochastic error sources acting on inertial sensors and determines the corresponding noise parameters. According to the noise parameters, the power spectral density (PSD) function of the stochastic error sources may be determined. An explanation of the PSD and AV may be found in [35]. The relationship between the AV and PSD of the noise parameters in the original data set *Ω*(*t*) is given by

(24) $$\sigma^{2}\left(\tau \right)=4\int_{0}^{\infty }S_{\Omega }\left(f\right)\frac{{sin}^{4}\left(\pi f\tau \right)}{{\left(\pi f\tau \right)}^{2}}df,$$

where *S_Ω_*(*f*) is the two-sided PSD of *Ω*(*t*).

The AV’s advantages may be described as time-domain analysis, noise source identification, and improved convergence [36]. Due to these advantages, the IEEE recommends the Allan Variance for identifying stochastic errors and determining noise types in inertial sensors.

### 2.5. GNSS Receiver Selection for GNSS/IMU System Design

The analysis of compact form factor GNSS modules for 10 DoF navigation system is conducted with three different GNSS modules as follows (Table 3):

- NEO-M9N or NEO-F10 from the u-blox company. This module supports UART and I2C communication interface, as well as NMEA and UBX protocols. This module was chosen as the base because u-blox is a leading manufacturer of GNSS modules and is compatible with ublox’s u-center application. The maximum receiver sensitivity is −165 dBm.
- MinewSemi ME32GR01. The module is pin-compatible with ublox’s NEO series and has the required sensitivity, low power consumption, and UART connectivity. The maximum receiver sensitivity is −165 dBm.
- ATGM332D of ZHONGKEWEI company. This low-cost module, pin-compatible with ublox’s NEO series, has the necessary sensitivity, low consumption, and connectivity via UART interface. The maximum receiver sensitivity is −162 dBm.

**Figure 6:**
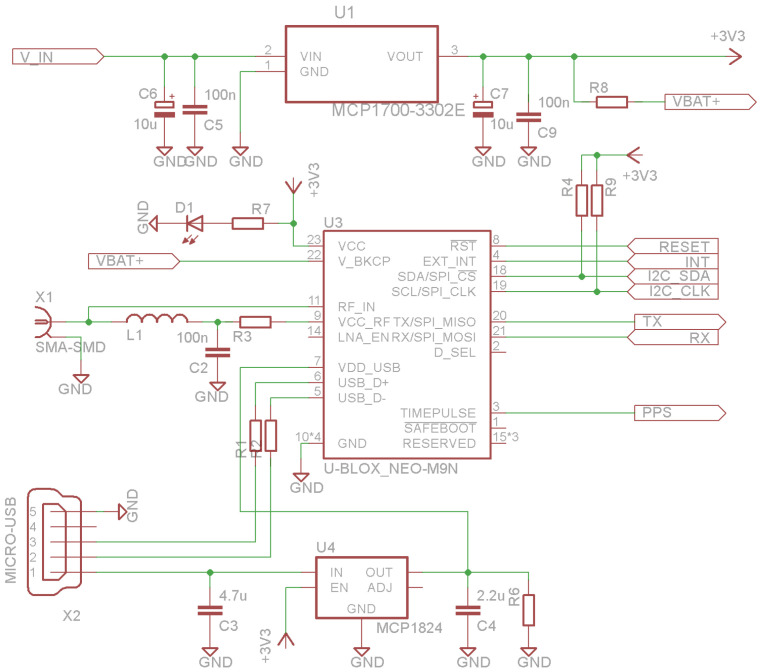
Schematic of the GNSS receiver.

The GNSS module is connected to the ESP32-C3 microcontroller via UART interface and I^2^C as an option, if supported (Figure 6). The data rate is set to 9600 bps or 38,400 bps depending on the selected receiver, which is powered by the main LDO regulator U1 or USB bus via the LDO regulator U4, only if the main power source is available. The GNSS receiver may use a passive or active antenna, which is powered from the VCC_RF pin via the low-pass filter (LPF) L1-C2 and current limiter resistor R3.

## 3. Results

### 3.1. The Noise Parameter Estimation of the Linear Accelerometers and the Angular Rate Sensors

The Kalman filter algorithm was implemented in the MATLAB environment (Version: 25.2.0.2998904 (R2025b)). The experimental algorithm verification was conducted in real-time situations on moving vehicles, and the obtained results were compared with similar angle values, which were calculated by both navigation systems.

**Figure 7:**
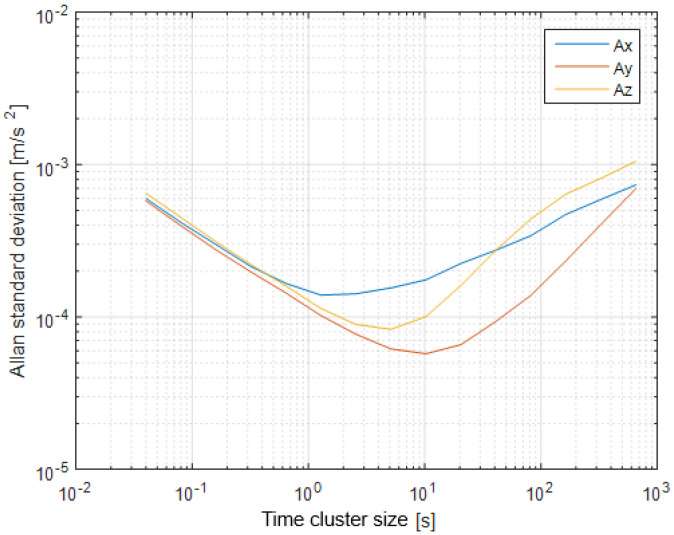
Allan Variance analysis of BMI270 linear accelerometer.

**Figure 8:**
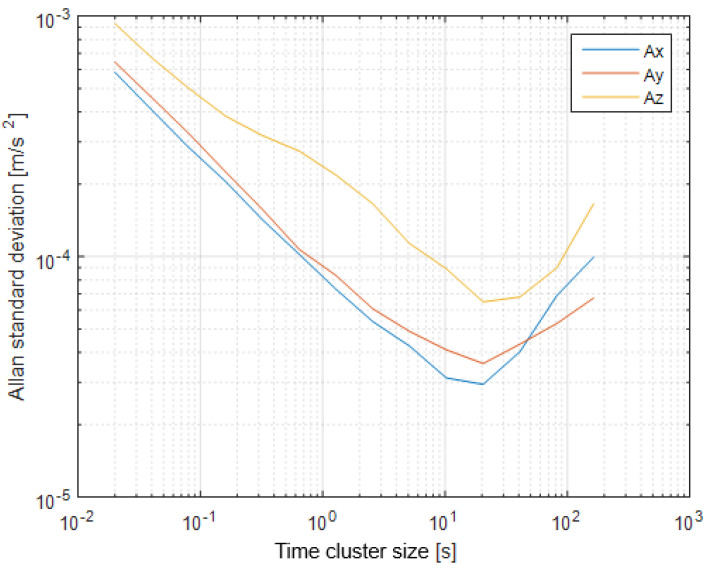
Allan Variance analysis of the ICM-42688-P linear accelerometer.

**Figure 9:**
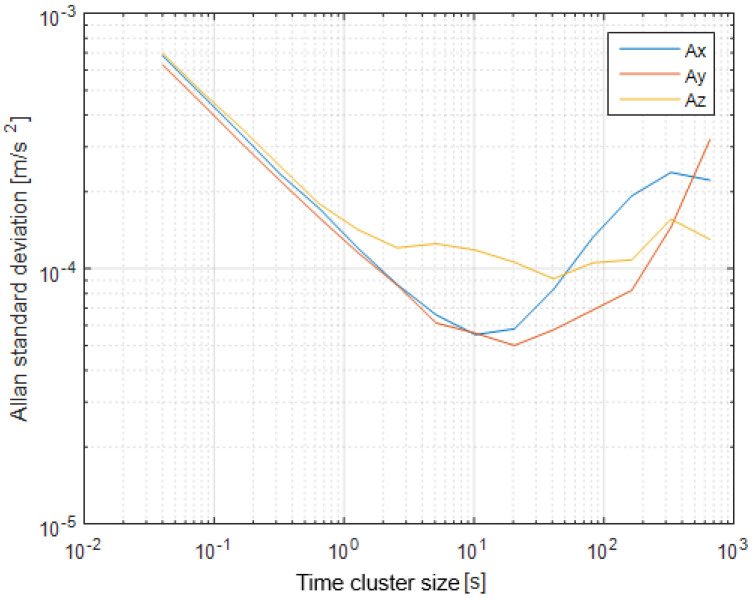
Allan Variance analysis of the LSM6DSL linear accelerometer.

**Figure 10:**
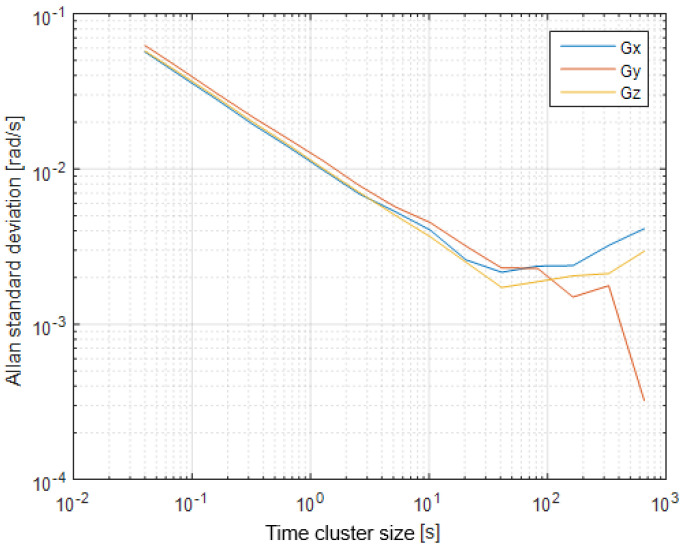
Allan Variance analysis of BMI270 angular rate sensor.

**Figure 11:**
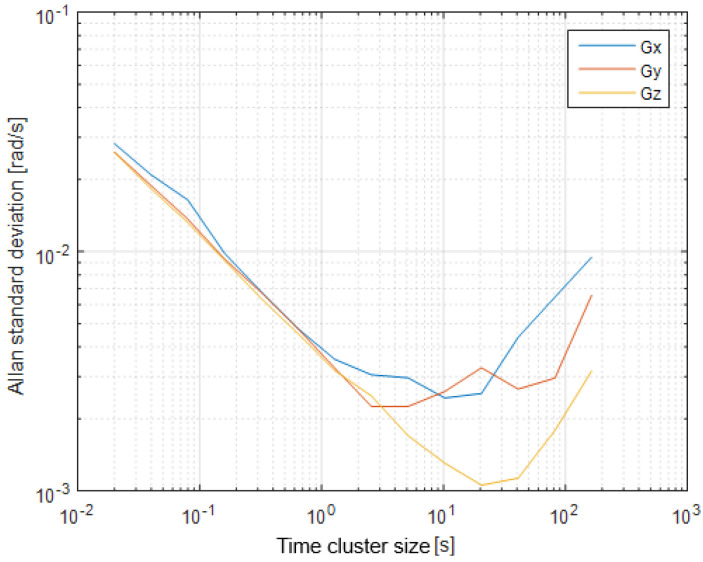
Allan Variance analysis of the ICM-42688-P angular rate sensor.

**Figure 12:**
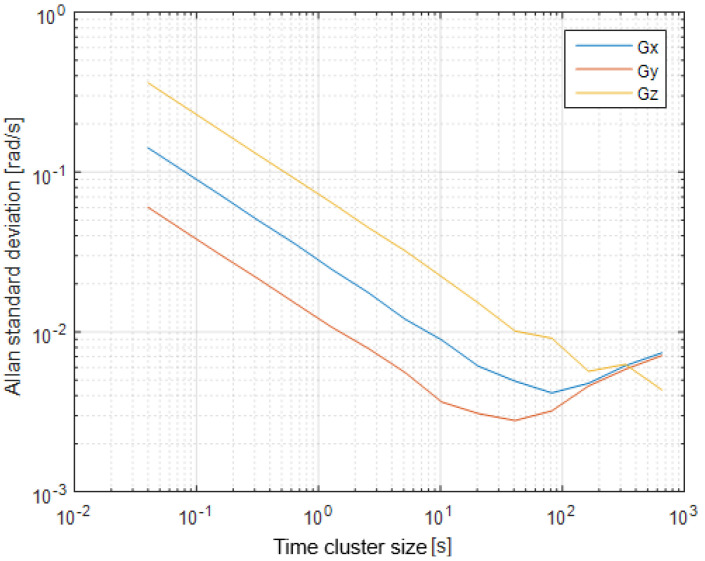
Allan Variance analysis of the LSM6DSL angular rate sensor.

**Figure 13:**
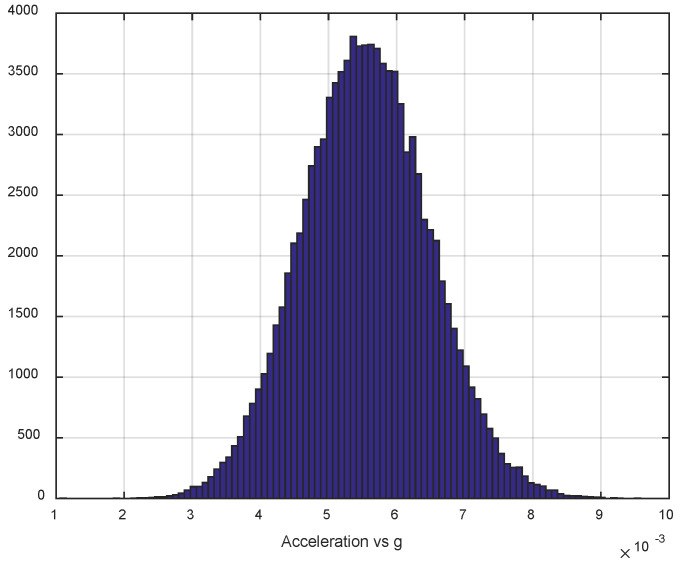
Histogram of the BMI270 accelerometer bias instability.

**Figure 14:**
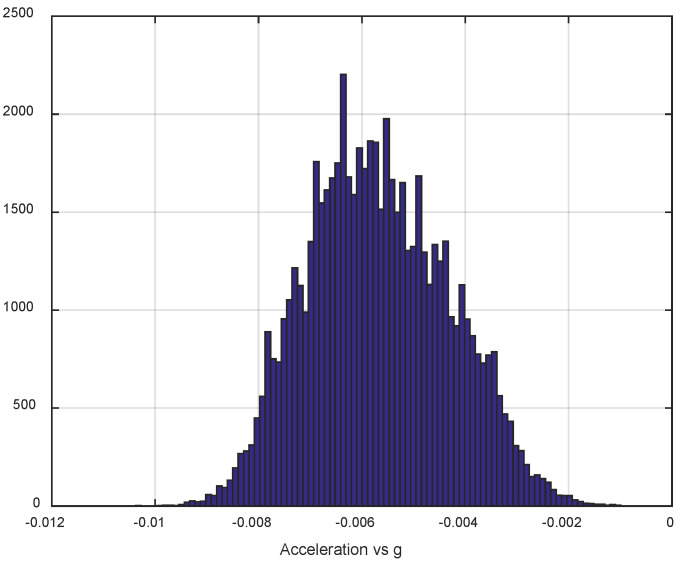
Histogram of the ICM-42688-P accelerometer bias instability.

**Figure 15:**
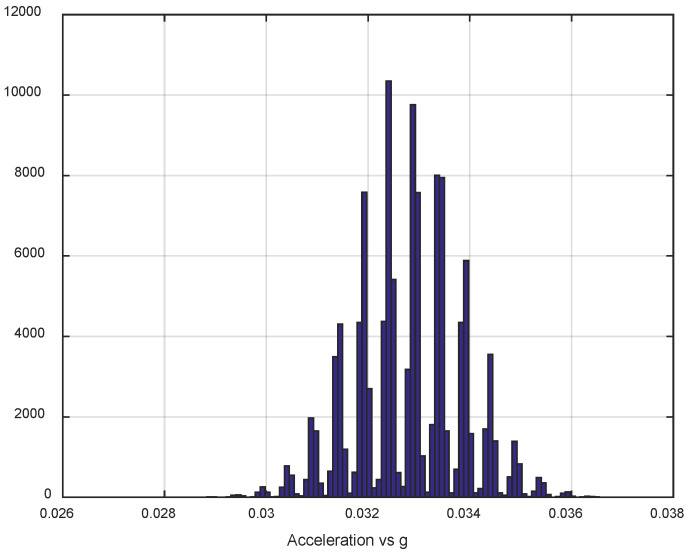
Histogram of the LSM6DSL accelerometer bias instability.

The first step of the test was determined by the calculation of the sensor noise parameters, i.e., the measurement noise covariance matrix R. The sampling rate was set to 50 Hz, which is equal to a time update period of 20 ms. The Allan Variance analyses of the linear accelerometers, shown in Table 3, are represented in Figure 7, Figure 8 and Figure 9 for the MEMS sensors BMI270, ICM-42688-P, and LSM6DSL, respectively. Figure 10, Figure 11 and Figure 12 represent the AV analysis of the gyroscopes of the same sensors, respectively, while Figure 13, Figure 14 and Figure 15 represent the histograms of the accelerometer bias instability for the corresponding sensors, in which the *y*-axis represents the number of the elements within each interval if the x values are divided to 1000 bins. The calculated coefficient values, according to the curve slope for the Allan Variance, are shown in Table 5, Table 6 and Table 7, respectively.

The parameters of the normal distribution of the sensor data are calculated and presented in Table 8, Table 9 and Table 10, respectively, using the MATLAB integrated function fitdist(), which estimates the mean (mu) and standard deviation (sigma), and the 95% confidence intervals for the parameters. The values are calculated by removing the mean value of the linear accelerations *ax*, *ay*, and *az*, to eliminate the influence of the sensor slope, while the total linear acceleration *A* is calculated by the raw *ax*, *ay*, and *az* values, according to the equation $A=\sqrt{a_{x}^{2}+a_{y}^{2}+a_{z}^{2}}$. All linear accelerations are normalized towards the standard acceleration of gravity on Earth’s surface *g* (Figure 13, Figure 14 and Figure 15).

The obtained values of σ_ax_, σ_ay_, σ_az_, σ_gx_, σ_gy_, and σ_gz_ for the MEMS sensor ICM-42688-P are placed in the measurement covariance matrix R (Equation (23)) and process noise covariance matrix Q (Equation (22)), and the initial Kalman filter values are configured.

### 3.2. GNSS Accuracy Estimation

**Figure 16:**
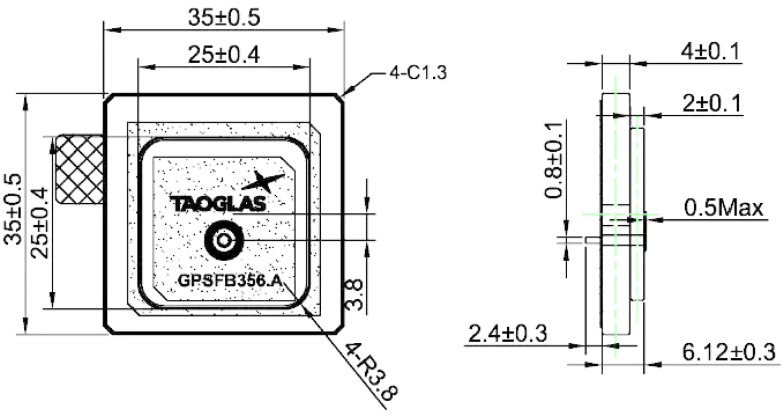
Taoglas GPSFB356.A GNSS antenna [37].

**Figure 17:**
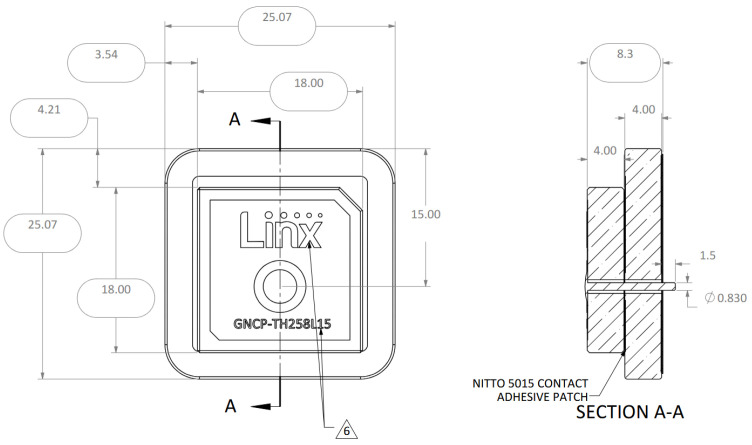
TE connectivity (Linx) ANT-GNCP-TH258L15 GNSS antenna [38].

**Figure 18:**
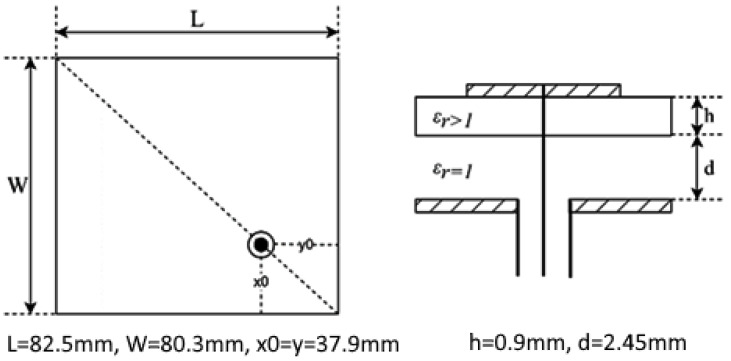
Circular polarized L1 patch antenna with suspended patch [39].

**Figure 19:**
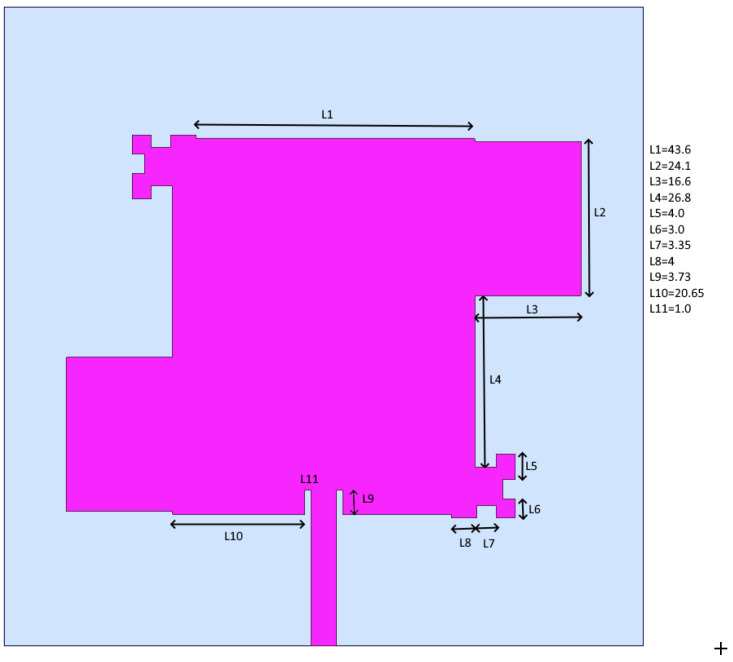
Dual band (L1/L5) fractal GNSS antenna [40]. All dimensions are in mm.

**Figure 20:**
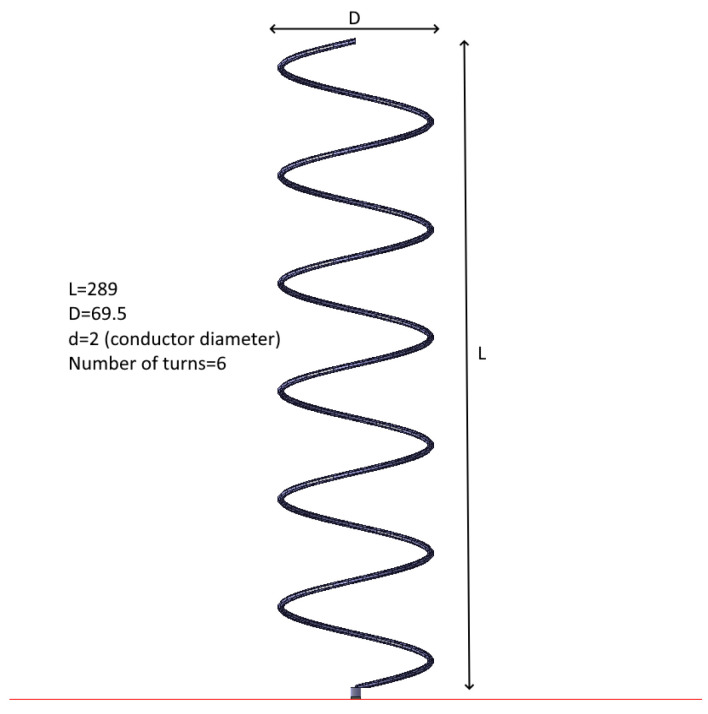
GNSS L1/L2/L5 helix antenna. All dimensions are in mm.

The GNSS accuracy estimation was tested using three different GNSS receivers, whose selection is discussed in Section 2.5. The tests were accomplished using five different antennas: two commercial ceramic patch antennas (Taoglas GPSFB356.A in Figure 16 and TE connectivity (Linx) ANT-GNCP-TH258L15 in Figure 17) and three designed patch ones, whose characteristics were previously described in [37,38]. The first proposed GNSS antenna was a circular polarized L1 patch antenna with suspended patch (Figure 18), the second one was a dual band (L1/L5) fractal GNSS antenna (Figure 19), and the third antenna was designed as a helix one (Figure 20). The antenna patterns are shown to explain the geometry of the used antennas and to compare the characteristics of the commercial antennas and the designed ones such as the antenna size, directivity, gain, etc., which affect the number of the visible satellites and the configuration of the tracking ones, used for the calculation of the position accuracy. All the proposed GNSS antennas were simulated and designed using Ansys EM Suite 2025R2 software (version 2025.2.0) from Ansys Inc., Canonsburg, PA, USA.

The GNSS performance was examined with a clear sky using U-center 2 evaluation software version 25.03.156221 from U-blox AG company (Thalwil, Switzerland). The analysis was accomplished via the connection of each antenna to the RF_IN input of the NEO-F10 u-blox AG GNSS receiver for 2–3 min. The position accuracy was analyzed according to the HDOP (Horizontal Dilution of Precision) and PDOP (Position Dilution of Precision) values, where lower HDOP values indicate better accuracy, while higher values suggest reduced reliability. A lower PDOP value helps a GNSS receiver obtain a fixed coordinate faster and maintain it for longer. The u-blox software calculates the Min/Max values, the averaged (Avg), and standard deviation (Std Dev) of the 2D coordinates. The satellite signal/position view and used/tracked satellites are also shown for comparison purposes.

**Figure 21:**
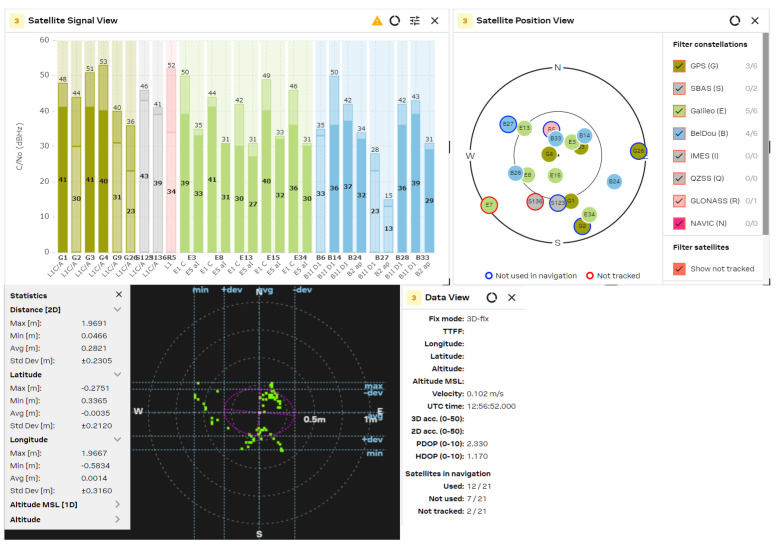
GNSS position analysis using the Taoglas GNSS antenna.

**Figure 22:**
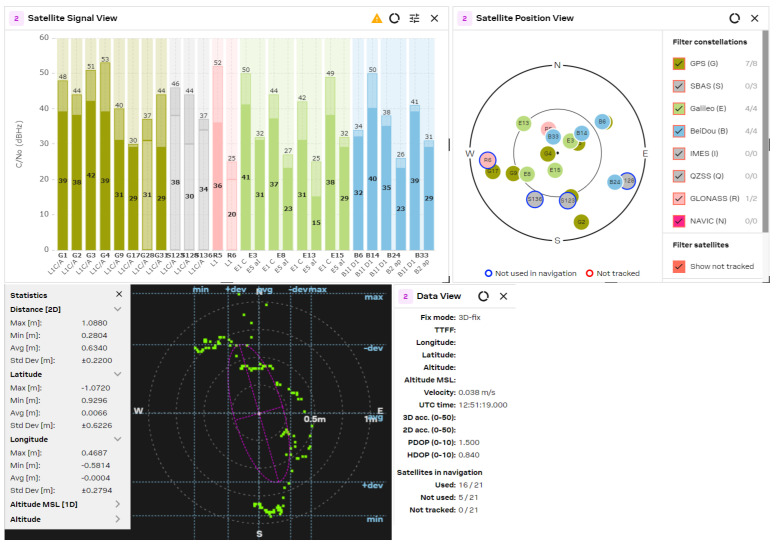
GNSS position analysis using the Lynx GNSS antenna.

**Figure 23:**
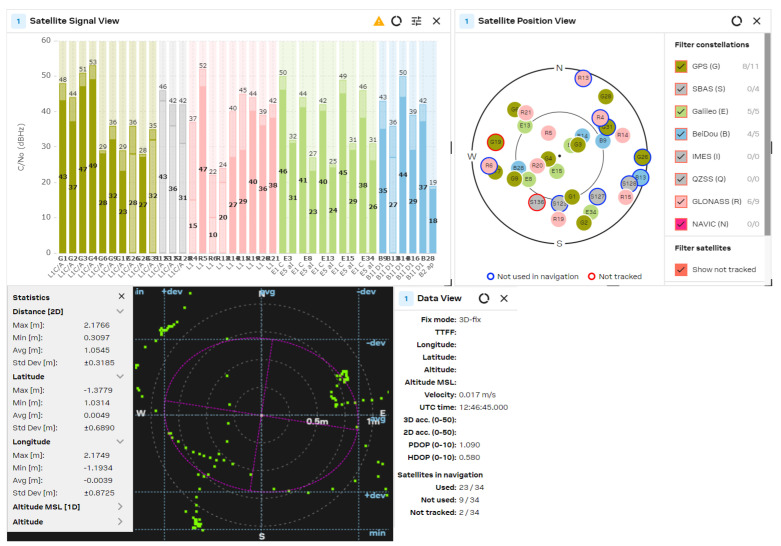
GNSS position analysis using the circular polarized L1 patch antenna with suspended patch.

**Figure 24:**
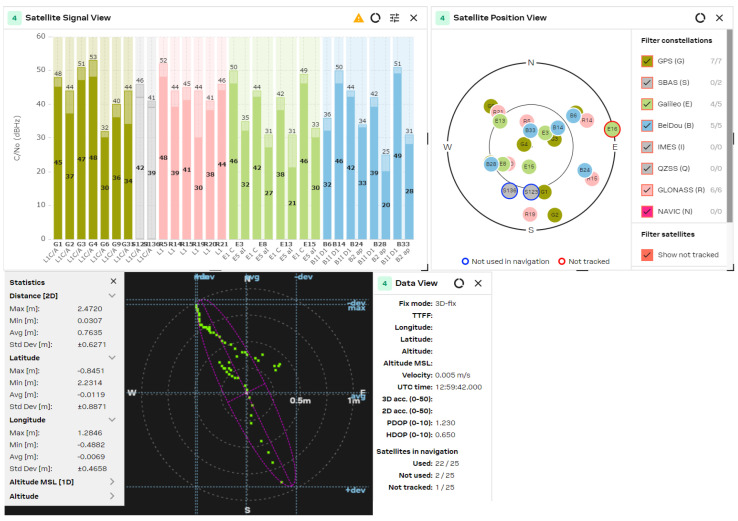
GNSS position analysis using the dual band (L1/L5) patch GNSS antenna.

**Figure 25:**
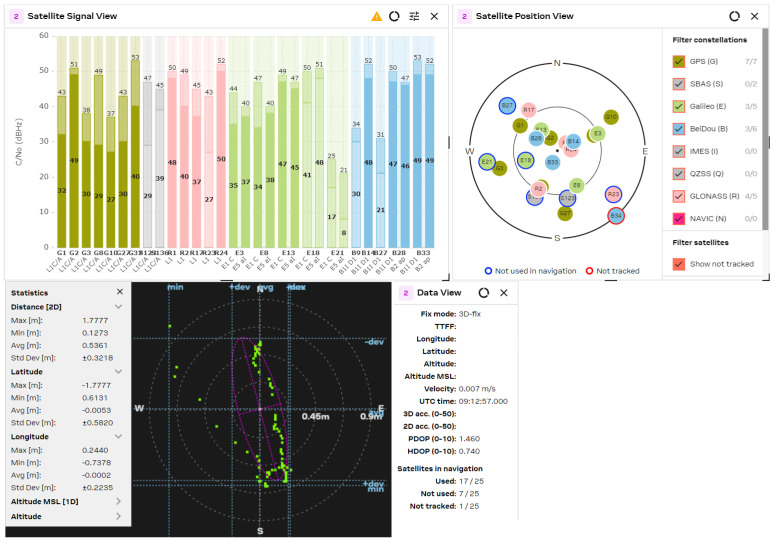
GNSS position analysis using the helix GNSS antenna.

The obtained results are shown in Figure 21, Figure 22, Figure 23, Figure 24 and Figure 25 for the five selected GNSS antennas, respectively.

**Figure 26:**
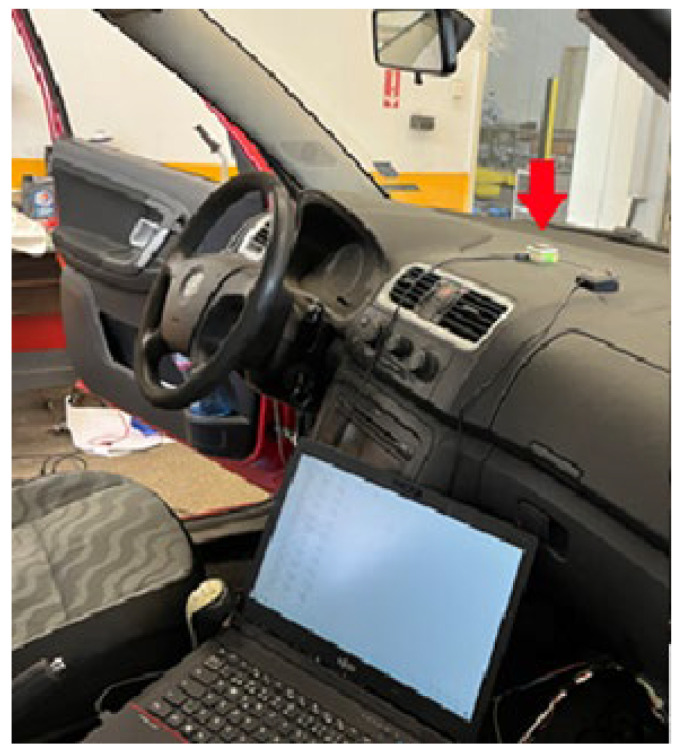
The system placement and installation.

An experiment was conducted by installing the proposed system on a Skoda Fabia II 1.4 TDI passenger car. The system was installed on the vehicle dashboard (the red arrow in Figure 26) to guarantee the best satellite view and smaller ferromagnetic influence over the magnetometer. The data were transferred to a PC via a USB connection and recorded on a hard disk as a TXT file using a serial terminal program @ 921,600 bps.

**Figure 27:**
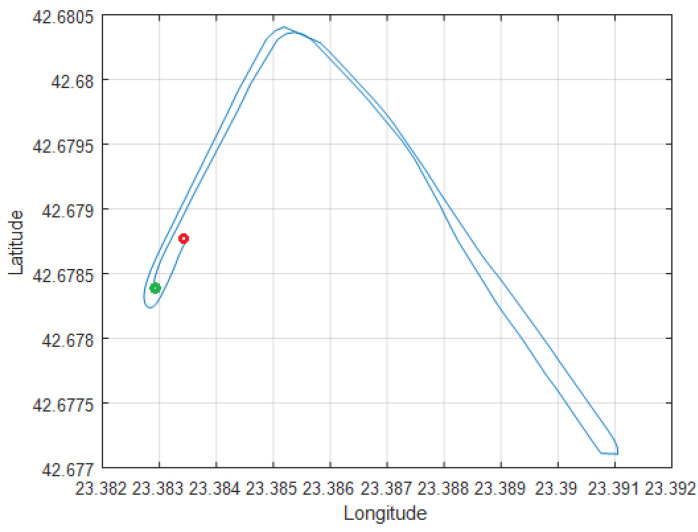
Test track (latitude/longitude).

The recorded data were imported in the MATLAB environment and were processed and synchronized to compare the linear and angular rate accelerations, magnetic field values, and vehicle position, speed, and course, which were calculated using the GNSS and IMU sub-systems. The recorded track is shown in Figure 27, where the green and the red dots show the starting and the final track points, respectively. The track curve was selected to contain 90- and 180-degree turns.

**Figure 28:**
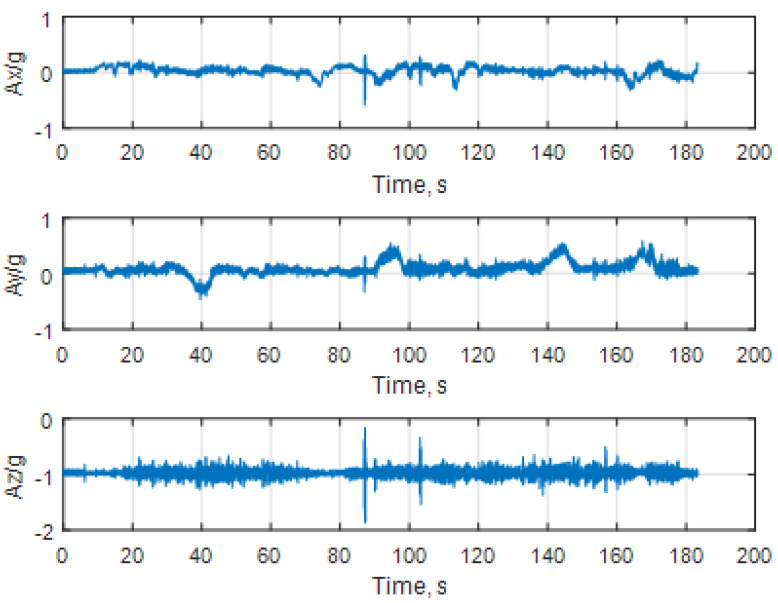
Representation of the linear accelerations normalized to the Earth’s gravity G.

**Figure 29:**
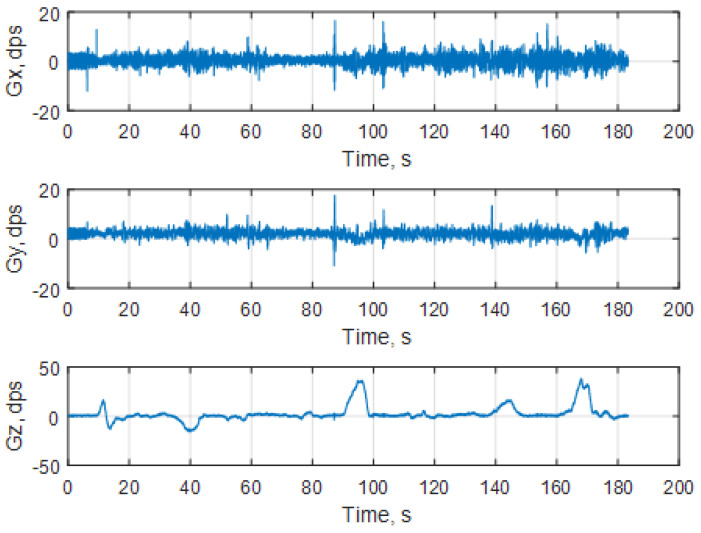
Representation of the angular rates in dps (degrees per second).

The measurement results from the linear accelerations and angular rates are shown in Figure 28 and Figure 29, respectively.

**Figure 30:**
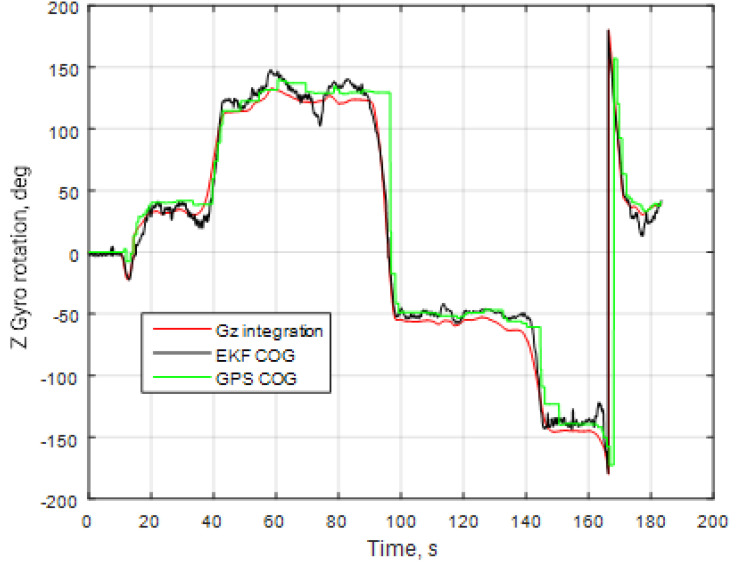
Comparison analysis of the heading angle calculation.

The obtained results may be used to correctly set the gyroscope sensor internal filters and calculate the engine rotation speed (after a Fourier analysis in the frequency domain), etc. The results from the different systems were compared regarding the heading angle *ψ* calculations. Their values were obtained using three independent sources: the Kalman filter output (KF COG), the GNSS receiver course over ground data (GNSS COG), and the angular rate sensor output integration (G_z_ integration) based on the trapezoidal method. The comparison results are shown in Figure 30, where the initial heading angle of all systems was set to 0. Since the GNSS and IMU data were sampled with different sampling rates (1 and 50 Hz, respectively), the GNSS data were linearly interpolated between the measurement points.

It is clear that the heading angle error between the Kalman filter output and other sources did not exceed 5°, and the Kalman filter output data are a bit noisy. Therefore, an additional smoothing filter can be added for a stable heading.

## 4. Discussion

The presented results show that GNSS and IMU systems may be used independently or as an integrated system to solve navigation problems regarding object location and spatial position. For example, the heading angle may be calculated using three independent sources, such as the angular rate gyroscope, magnetometer, and GNSS receiver. The study of the noise parameters of the MEMS sensors shows that the commercial linear acceleration sensors have approximately similar characteristics, such as a digital resolution of 16 bits, a measurement range up to 16 g, a zero-rate offset in the range from ±10 to ±20 mg, a noise density of 70 µg/√Hz, and ODR values of ones to tens of kHz. At the same time, the angular rate sensors are distinguished by higher noise levels of 8 mdps/√Hz at the same ODR values. This circumstance was estimated during calculation of the Allan Variance deviation calculations represented in Figure 7, Figure 8, Figure 9, Figure 10, Figure 11, Figure 12 and Figure 13. The theoretical analysis of the MEMS sensor noise parameters enabled the selection of the ICM-42688-P sensor on the basis of its higher digital resolution of up to 19 bits, higher ODR of up to 32 kHz, and very low angular rate sensor noise density of 2.8 mdps/√Hz. The experimental study of the Allan Variance deviation confirmed this, as shown in Figure 7, Figure 10 and Figure 13. The calculated values of the Allan Variance deviation for the linear accelerometers showed similar values for all the tested sensors (Figure 9, Figure 12 and Figure 15). The calculated normal distribution parameters showed that the sigma value was equal to appr. 10^−3^ for all the tested linear accelerometers. However, the analysis of the angular rate sensor characteristics showed that the mu and sigma values were equal to 10^−1^ ÷ 10^−2^ for the LSM6DSL sensor and 10^−1^ for the BMI270, while the corresponding values were lower by one order for the ICM-42688-P sensor.

The analysis of the GNSS system performance showed excellent accuracy, due to the very low HDOP values for all selected antennas below 1.2. The worst results were obtained using ceramic antennas (PDOP = 1.5 ÷ 2.4, HDOP = 0.8 ÷ 1.2), while the corresponding values with helix and patch antennas were PDOP = 1.0 ÷ 1.5 and HDOP = 0.58 ÷ 0.74. This situation was expected, as the patch antennas have a wider directivity diagram, lower losses, and good phase characteristics in the L1/L5 GNSS frequency ranges. Their disadvantage is a larger size, which is not critical in transport systems, for example. In all cases, the position accuracy was in the sub-meter range due to the SBAS Differential Correction Service corrections. The number of visible satellites from all satellite constellations exceeded 20 in all cases and can reach values up to 35 in open sky scenarios.

## 5. Conclusions

This study examined a system for the estimation of object location and spatial position, based on a GNSS receiver and a 10 DoF IMU system. The calculations were performed using two Kalman filters: one for the attitude and heading estimation and the second for GNSS/IMU integration. This may be used in dual core microcontrollers such as the ESP32-S3 to implement faster calculations. Future work will be directed towards the second Kalman filter, which will be used for dead-reckoning purposes.

## Figures and Tables

**Table 1:** Linear accelerometer sensor parameter comparison.

	BMI270 [18]	ICM-42688-P [19]	LSM6DSR [20]
Digital resolution	16-bit	16–18-bit	16-bit
Measurement range	±2 g–±16 g	±2 g–±16 g	±2 g–±16 g
Zero-rate offset	±20 mg	±20 mg	±10 mg
Noise density	160 µg/√Hz	70 µg/√Hz	60 µg/√Hz
Output data rate (ODR)	12.5 Hz–1.6 kHz	1.5625 Hz–32 kHz	1.6 Hz–6.667 kHz

**Table 2:** Angular rate sensor parameter comparison.

	BMI270	ICM-42688-P	LSM6DSR
Digital resolution	16-bit	16–19-bit	16-bit
Measurement range	±125 dps–±2000 dps	±15.6 dps–±2000 dps	±15.6 dps–±4000 dps
Zero-rate offset	±0.5 dps	±0.5 dps	±1 dps
Noise density	8 mdps/√Hz	2.8 mdps/√Hz	5 mdps/√Hz
Output data rate (ODR)	25 Hz–6.4 kHz	12.5 Hz–32 kHz	12.5 Hz–6.667 kHz

**Table 3:** PCB configuration of sensors.

	Board #1	Board #2	Board #3
**GNSS receiver**	NEO-F10	ATGM332D	ME32GR01
**Accelerometer and gyroscope**	ICM-42688-P	BMI270	LSM6DSR
**Magnetometer**	MMC5983MA	MMC5983MA	MMC5983MA
**Barometer**	LPS22HB	LPS22HB	LPS22HB

**Table 4:** Sensor output data rate supported.

	Barometer	Accelerometer	Gyroscope	Magnetometer
Supported output data rate, Hz	1	12.5	12.5	50
	10	25	25	100
	25	50	50	225
	50	100	100	580
	75	200	200	1000
		500	500	
		1000	1000	
		2000	2000	
		4000	4000	
		8000	8000	
		16,000	16,000	
		32,000	32,000	

**Table 5:** Coefficient values according to the curve slope for the Allan Variance of the BMI270 sensor.

		Coefficient Value	Coefficient Value	Coefficient Value
	**Curve Slope**	**Ax, m/s^2^**	**Ay, m/s^2^**	**Az, m/s^2^**
**Q**	−1	1.79 × 10^−5^	1.71 × 10^−5^	1.93 × 10^−5^
**N**	−0.5	1.18 × 10^−4^	1.10 × 10^−4^	1.24 × 10^−4^
**B**	0	1.38 × 10^−4^	5.66 × 10^−5^	8.03 × 10^−5^
**K**	0.5	1.77 × 10^−5^	1.35 × 10^−5^	2.55 × 10^−5^
**S**	1	6.78 × 10^−7^	9.00 × 10^−7^	9.74 × 10^−7^
		**Gx, rad/s**	**Gy, rad/s**	**Gz, rad/s**
**Q**	−1	1.60 × 10^−3^	1.80 × 10^−3^	1.60 × 10^−3^
**N**	−0.5	1.11 × 10^−2^	1.23 × 10^−2^	1.15 × 10^−2^
**B**	0	1.90 × 10^−3^	9.47 × 10^−4^	1.80 × 10^−3^
**K**	0.5	6.51 × 10^−5^	3.13 × 10^−5^	1.08 × 10^−4^
**S**	1	2.18 × 10^−6^	1.04 × 10^−6^	4.52 × 10^−6^

**Table 6:** Coefficients according to the curve slope for the Allan Variance of the ICM-42688-P sensor.

		Coefficient Value	Coefficient Value	Coefficient Value
	**Curve Slope**	**Ax, m/s^2^**	**Ay, m/s^2^**	**Az, m/s^2^**
**Q**	−1	1.64 × 10^−5^	1.81 × 10^−5^	2.62 × 10^−5^
**N**	−0.5	1.16 × 10^−4^	1.24 × 10^−4^	1.86 × 10^−4^
**B**	0	3.18 × 10^−5^	2.46 × 10^−5^	6.50 × 10^−5^
**K**	0.5	3.60 × 10^−6^	1.09 × 10^−6^	6.40 × 10^−6^
**S**	1	2.10 × 10^−7^	4.69 × 10^−8^	4.56 × 10^−7^
		**Gx, rad/s**	**Gy, rad/s**	**Gz, rad/s**
**Q**	−1	8.11 × 10^−4^	7.38 × 10^−4^	7.34 × 10^−4^
**N**	−0.5	5.40 × 10^−3^	5.20 × 10^−3^	5.10 × 10^−3^
**B**	0	2.00 × 10^−3^	1.80 × 10^−3^	9.07 × 10^−4^
**K**	0.5	2.86 × 10^−4^	2.27 × 10^−4^	1.23 × 10^−4^
**S**	1	1.31 × 10^−5^	1.88 × 10^−5^	1.11 × 10^−5^

**Table 7:** Coefficient values according to the curve slope for the Allan Variance of the LSM6DSL sensor.

		Coefficient Value	Coefficient Value	Coefficient Value
	**Curve Slope**	**Ax, m/s^2^**	**Ay, m/s^2^**	**Az, m/s^2^**
**Q**	−1	1.91 × 10^−5^	1.77 × 10^−5^	1.98 × 10^−5^
**N**	−0.5	1.35 × 10^−4^	1.25 × 10^−4^	1.40 × 10^−4^
**B**	0	5.39 × 10^−5^	4.73 × 10^−5^	1.02 × 10^−4^
**K**	0.5	2.93 × 10^−6^	6.11 × 10^−6^	7.17 × 10^−6^
**S**	1	9.00 × 10^−8^	4.58 × 10^−7^	2.40 × 10^−7^
		**Gx, rad/s**	**Gy, rad/s**	**Gz, rad/s**
**Q**	−1	4.00 × 10^−3^	1.70 × 10^−3^	1.03 × 10^−2^
**N**	−0.5	2.59 × 10^−2^	1.20 × 10^−2^	6.89 × 10^−2^
**B**	0	2.60 × 10^−3^	1.50 × 10^−3^	3.70 × 10^−3^
**K**	0.5	9.01 × 10^−5^	4.55 × 10^−5^	1.63 × 10^−4^
**S**	1	3.09 × 10^−6^	1.39 × 10^−6^	6.38 × 10^−6^

**Table 8:** Calculated normal distribution parameters of the BMI270 sensor.

	Mu	Sigma
Ax	-	1.41 × 10^−3^
Ay	-	1.21 × 10^−3^
Az	-	1.76 × 10^−3^
Gx	1.08 × 10^−1^	7.99 × 10^−2^
Gy	2.19 × 10^−1^	8.86 × 10^−2^
Gz	−1.46 × 10^−1^	8.18 × 10^−2^
A	5.55 × 10^−3^	9.32 × 10^−4^

**Table 9:** Calculated normal distribution parameters of the ICM-42688-P sensor.

	Mu	Sigma
Ax	-	8.35 × 10^−4^
Ay	-	9.11 × 10^−4^
Az	-	1.38 × 10^−3^
Gx	−3.76 × 10^−3^	4.26 × 10^−2^
Gy	7.52 × 10^−3^	3.85 × 10^−2^
Gz	−2.92 × 10^−2^	3.72 × 10^−2^
A	−5.70 × 10^−3^	1.40 × 10^−3^

**Table 10:** Calculated normal distribution parameters of the LSM6DSL sensor.

	Mu	Sigma
Ax	-	1.02 × 10^−3^
Ay	-	9.83 × 10^−4^
Az	-	1.04 × 10^−3^
Gx	1.54 × 10^−1^	2.01 × 10^−1^
Gy	−3.72 × 10^−1^	8.58 × 10^−2^
Gz	2.44 × 10^−2^	5.13 × 10^−1^
A	3.28 × 10^−2^	1.05 × 10^−3^

## Data Availability

The data that support the findings of this study are available at public service: https://doi.org/10.5281/zenodo.17475705.
